# Synthesis, Characterization and Anti-Inflammatory Activity of Some 1, 3,4 -Oxadiazole Derivatives

**Published:** 2013

**Authors:** Arvind Kumar Singh, M Lohani, R Parthsarthy

**Affiliations:** a *Kamla Nehru Institute of Management and Technology, Faculty of Pharmacy, Sultanpur up, India. *; b *Integral University Luck now, Uttarpradesh India.*

**Keywords:** 1, 3, 4-Oxadiazoles, Anti-inflammatory, Synthesis, Heterocyclic

## Abstract

A series of five-membered heterocyclic rings were synthesized by the reaction between benzoyl chloride and various chlolro-nitro-benzoyl chlorides and semi carbazide to form (C_1_- C_7_) compounds and was tested for their anti-inflammatory activity determined by rat-paw-oedema method. All the synthesis compounds have been characterized by ^1^HNMR, IR and Mass spectral data. The compounds were purified by column chromatography. All synthesized derivatives were determined by the carrageenan-induced rat-paw-oedema model for anti-inflammatory activity. The entire compound gives good response for the anti-inflammatory activity: [3-Chloro-*N*-[5-(3-Chloro-phenyl)-[1,3,4] oxadiazole-2yl] benzamide (C_4_), and [4-Nitro-*N*-[5-(4-Nitro-phenyl)-[1,3,4] oxadiazole-2yl] benzamide (C_7_). For this activity, indometacin was used as a standard drug and compared to new synthesized drugs. Some new synthesized drugs have shown better activities for the anti-inflammation.

## Introduction

1, 3, 4-oxadiazole derivatives are heterocyclic compounds containing one oxygen and two nitrogen atoms in a five-membered-ring. 1,3,4-oxadiazole derivatives have played a major role in the pharmaceutical chemistry. The number of so many synthetic compounds with oxadiazole nucleus used for antibacterial (1-5), antifungal (6-9), analgesic and anti-inflammatory activities (10-13). Derivatives of 1,3,4-oxadiazole with suitable substitution at 2,5-position have already been reported to have possible biological activities. 1,3,4-oxadiazole derivatives act as anticonvulsant and diuretics (14). These observations and our interest in the pharmaceutical chemistry of heterocyclic compounds promoted us to have synthesized different derivatives of 1,3,4-oxadiazole with different substituent at 2 and 5-positions. These derivatives have been also screened for their anti-inflammatory activity. Mostly, five-membered-ring aromatic systems having three heteroatoms at symmetrical position have been studied because of their physiological properties (15-16). It is also well established that various derivatives of 1,3,4-oxadiazole exhibit broad spectrum of pharmacological properties such as antibacterial and antifungal activities (17-18). 1,3,4-oxadiazole showed antibacterial properties similar to those of well known sulphonamide drugs (19). 

## Experimental

All chemicals were supplied by (Merck and S.D fine chemicals Lucknow India). Melting point (m.p) was determined by open capillary tube method. Purification of compounds was checked by column chromatography and silica gel-G (60-120 mess) and silica gel GF_254 _(4:1) for preparation of TLC plates and also used the solvent system 5% ethyl acetate in pet. Ether and spots were seen under iodine vapours and UV light chamber. IR spectra were obtained on a Perkin-Elmer 1720 FT-IR-Spectrometer (KBr-sol^n^/pellets). ^1^HNMR spectra were noted in Brucker Ac-400 MHz spectrometer using TMS as internal standard in DMSO/CDCl_3_ and mass spectra (m/z %) recorded by VG ZAB-HS (FAB) instrument.

*Material and methods*

General procedure for synthesis of compounds (C_1_ to C_7_ ):- (1:1 molar ratio) Aromatic, phosphorus pentachloride and benzene were taken in RBF, fitted with air condenser and calcium chloride guard tube. The mixture was heated gently to melt with vigorous shaking at around 50°C. After 30 min excess POCl_3_ was distilled out. The residue was dried well and used for the next reaction. Then, semicarbazide was added to the respected acid chloride and reflux for 5 h. These programs of the reaction were monitored by checking the TLC. The excess benzene was distilled out, neutralizing with aq. NaHCO_3_ and the compound was extracted with chloroform. The crude was obtained through distillation of chloroform under reduce pressure

*Anti-inflammatory activity* ^20^

Anti-inflammatory activity of all synthesized derivatives was determined by the carrageenan-induced rat paw oedema model. Albino rats (100-200 g) were divided into 3 groups as control, test and standard (six animals per group). Overnight fasted animals were used and during that period only tap water was given. Generally, indomethacin was used as standard drug. Both test and standard drugs were suspended in 1% carboxymethyl cellulose (CMC) and administered orally through gastric gavage needle. One percent of CMC was administered in control group. After 1 h of administrating the compound, we induced the carrageenan (1%) by the sub planner surface of the right hind paws of animals. The initial paw volume and also the paw volume after 3 and 6 h of administrating carrageenan were measured. Percent paw oedema inhibition was calculated.

## Results and Discussion

At the end of the experiment, it has been concluded that the compounds synthesized in the project have good yield value. The synthesized oxadiazole compounds were identified and characterized by IR, ^1^H NMR and MASS spectra. Then, the pharmacological activity was done. The entire compound had a good response for Anti-inflammatory activity : [3-Chloro-N-[5-(3-Chloro-phenyl)-[1,3,4] oxadiazole-2yl]benzamide (C_4_),and [4-Nitro-N-[5-(4-Nitro-phenyl)-[1,3,4] oxadiazole-2yl]benzamide (C_7_). Substitution of 2-chloro-benzoic acid, at 2,5- position anti-inflammatory activity greater (C_2_) than 3-chloro substituted compound (C_3_)and 4-cloro-benzoic acid compound (C_4_) substituted at 2,5 position anti-inflammatory activity greater than 2-chloro-substituted compound (C_2_). While substitution of 4-nitro-compounds (C_7_) at 2,5-position greater than other 2-nitro and 3-nitro substituted compounds (C_5_ and C_6_).

*Compound 1: [N-(5-Phenyl-[1, 3, 4] oxadiazol-2-yl)-benzamide]*

IR( KBr,cm-1) : 3214( NH) ,1664(C=O) , 1070( N-N) ,1232(C-O-C) ; ^1^HNMR(DMSO-ds,400 M Hz), 8.72 (s, 1H, J = 7.6 Hz) , 7.72 (d, 3H, J = 7.9 Hz), 7.82 (d, 1H, J = 7.9 Hz), 7.62 (d, 1H, J = 7.6 Hz, MASS (ESI):m/z (%), 266 (23), 262 (14), 260 (100), 249 (17), 248 (100). analytical calculated for C_15_H_11_N_3_O_2_ c = 67.90, H = 4.23, N = 15.86, O = 12.12, found = C = 67.92, H = 4.15, N = 15.84, O = 12.07.

*Compound 2: [2-Chloro-N-[5-(2-chloro-phenyl)-[1,3,4]oxadiazol-2-yl]benzamide]*

IR( KBr,cm-1): 3270(NH),1670( C=O) ,1072( N-N)1240( C-O-C),776( C-Cl) ;1HNMR (DMSO-ds, 400 MHz) ,7.96 (d, 1H, J = 7.5) ,7.78 (d, 1H, J = 7.4) ,7.72 (d, 2H, J = 8.87), 7-7.8 (m, 3H, J = 8.2); MASS (CSM), m/z (%), analytical calculated for C_15_H_9_N_3_O_2_Cl_2_; C = 49.60, H = 2.96, N = 23.48. found C = 49.62, H = 2’86, N = 23.44.


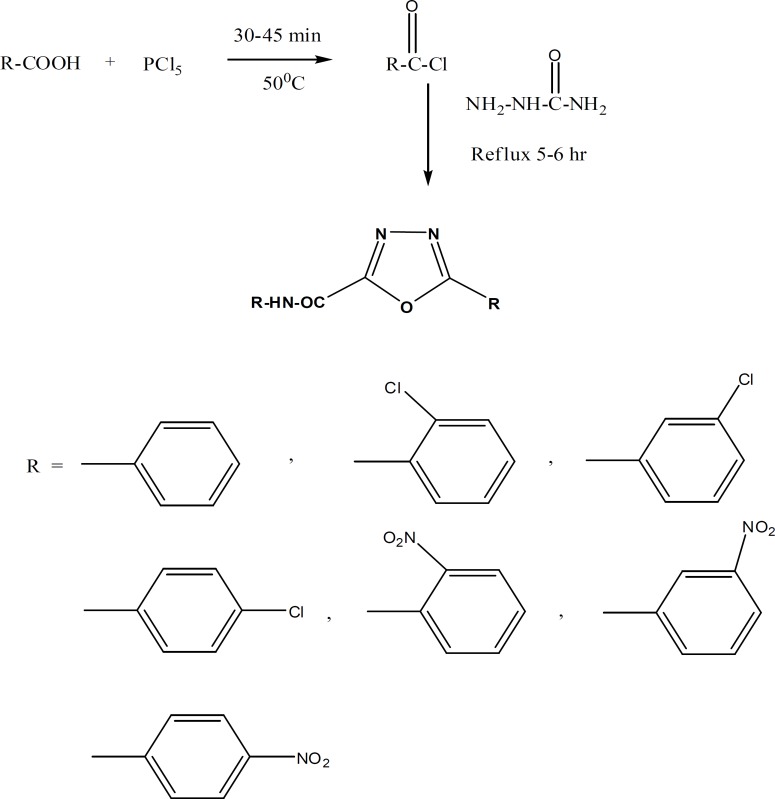


*Compound 3: [4-Chloro-N-[5-(4-chloro-phenyl)-[1,3,4] oxadiazol-2-yl]-benzamide]*

IR (KBr , cm-1) P: 3272 ( NH) , 1668 (C=O), 1076 (N-N), 1242 (C-O-C), 778 (C-Cl); 1HNMR (DMSO-ds,400 MHz), 7.76 (d, 2H, J = 7.3 Hz), 7.68 (d, 1H, J = 7.2 Hz), 7.73 (d, 2H, J = 8.2 Hz),7.78 (m, 3H, J = 8.32 Hz); MASS (C-SI), m/z (%) analytical calculated for C15H9N3O2Cl2, C = 48.98, H = 2.83, N = 23.48, found, C = 47.96, H = 2.81, H = 23.32. 

*Compound 4: [3-Chloro-N-[5-(3-Chloro-phenyl)-[1,3,4] oxadiazole-2yl]benzamide*

IR (KBr, cm-1): 3268 (NH), 1668 (C=O), 1072 (N-N), 1242 (C-O-C), 725 (C-Cl); 1HNMR (DMSO, ds, 400 MHz) ,7.72-7.75 (d, 2H, J = 8.4 Hz), 7.78 (m, 3H, J = 8.32 Hz). MASS (CSM); M\Z%-Anal calculator for C15H9N3O2Cl2, C = 48.41, H = 2.79, N = 23.25, Found C = 47.98, H = 2.76; N = 23.16. 

*Compound 5: [2-Nitro-N-[5-(2-Nitro-phenyl)-[1, 3, 4] oxadiazole-2yl] benzamide*

IR (KBr cm-1) :- 3272 (NH), 1670 (C=O), 1078 (N-N), 1260 (C-O-C), 780 (C-Cl) H1NMR (DMS+ds400M H2):- 7.70 (d, 1H, J = 7.25 Hz), 7.78 (d, 1H, J = 7.4 Hz), 7.78 (d, 2H, J = 8.1 Hz), 7.25 (m, 3H, J = 8.21 Hz) Mass (CSM) (M/Z (%)-Anal calculator for C14H9N5O6, C = 48.97, H = 2-93, N = 30.48, formed C = 48.27, N = 2.90, N = 30.42. 

*Compound 6: [3-Nitro-N-[5-(3-Nitro-phenyl)-[1,3,4] oxadiazole-2yl]benzamide*

IR (KBr Cm-1):- 3271(NH), 1668(C=O), 1070(N-H), 1265(C-O-C), 786(C-Cl) H1NMR (DMS+ds400M H2):- 7.71(d, 1H, J = 7.24 Hz), 7.77(d, 1H, J = 7.3 Hz), 7.73 (d, 1H, J = 8.3 Hz), 7.22 (m, 1H, J = 8.21 Hz), Mass,(CSM) M/Z (%)-Anal calculator for C14H9N5O6 C = 48.92, H = 2.44, N = 30.47, found C = 48.46, H = 2.90, N = 30.45.

*Compound 7: [4-Nitro-N-[5-(4-Nitro-phenyl)-[1, 3, 4] oxadiazole-2yl] benzamide*

IR(KBr Cm-1) :- 3272(NH), 1665 (C=O), 1078 (N-N), 1260 (C-O-C), 783(C-Cl) H1NMR (DMS+ds400M H2):- 7.70(d, 1H, J = 7.23 H2), 7.2 (d, 1H, J = 7.2 H2), 7.74 (d, 1H, J = 8.4 H2), 7.26 (m, 1H, J = 8.20 H2), Mass, (CSM) M/Z(%)-Anal calculator for C14H9N5O6 C = 48.98, H = 292, H = 30.46, formed C = 48.92, H = 2.48, N = 30.36.

**Table 1:** Physical properties of compounds (C_1_ to C_7_).

**Compounds**	**Yield (%)**	**Rf**	**MP(°C)**	**Mol. Formula**	**Mol. Wt.**
C_1_	72%	0.715	212	C_15_H_11_N_3_O_2_	265.2
C_2_	66%	0.692	214	o-C_15_H_9_N_3_O_2_Cl_2_	334.16
C_3_	79%	0.678	213	m-C_15_H_9_N_3_O_2_Cl_2_	334.16
C_4_	82%	0.682	211	p-C_15_H_9_N_3_O_2_Cl_2_	334.16
C_5_	80%	0.721	273	o-C_15_H_9_N_5_O_6_	355.26
C_6_	73%	0.761	266	m-C_15_H_9_N_5_O_6_	355.26
C_7_	78%	0.672	271	p-C_15_H_9_N_5_O_6_	355.26

**Table 2:** Anti-inflammatory activities of compounds C_1_ to C_7_

**Columns**	**Dose Mg/Kg**	**Inhibition of paw oedema after 3 h (%)1**	**Inhibition of paw oedema after 6 h (%)2**
C-1	30	3.28 ± 0.28	58.24
C-2	30	2.48 ± 0.23	56.48
C-3	30	3.46 ± 0.22	51.16
C-4	30	1.62 ± 0.27	70.98
C-5	30	3.26 ± 0.241	59.48
C-6	30	3.22 ± 0.281	53.98
C-7	30	1.52 ± 0.271	69.54
Control	_	0 .36 ± 0.28	_
Indomethacine	40	1.78 ± 0.340	66.44

1: Dose for 1-7: 30 mg/Kg b.wt; 2: Dose for indomethacin 40 mg/Kg b.wt; mean ± SEM; n+6

## References

[1] Sahin G, Palaska E, Ekizoglu M, Ozalp M (2002). Synthesis and antimicrobial activity of some 1,3,4-oxadiazole derivatives II. Farmaco.

[2] Chaudhary M (2001). Synthesis and pharmacological activity of some 1,2,4 oxadiazole I. Journal Chem.

[3] Hiremath SP, Sonar VN, Sekhar KR, Purohit MG (1989). Synthesis and antibacterial activity of 1,3,4, oxadiazole. Indian J. Chem.

[4] Mashooq A Bhat, Khan SA, Siddiqui N (2005). Synthesis and antibacterial activity of coumarin incorporated 1,3,4, oxadiazole. Indian J. Heterocyclic Chem.

[5] Priya V Frank, Balakrishna Kalluraya (2005). The chemistry of imidazole derivatives. Indian J. Chem.

[6] Hansong Chen, Zhengming Li, Yufeng Han (2000). Synthesis and Fungicidal Activity against Rhizoctonia solani of 2-Alkyl (Alkylthio)-5-pyrazolyl-1,3,4-oxadiazoles (Thiadiazoles). J. Agric. Food Chem.

[7] Xia-Juan Zou, Lu-Hua Lai, Gui-Yu Jin, Zu-Xing Zhang (2002). Synthesis and antimicrobial activities if 1,3,4 oxadiazole derivatives. J. Agric Food Chem.

[8] Saad H, Indian J Chem (1996). Determination of ranitidine in pharmaceutical preparations using manual and flow injection potentiometry and spectrophotometry. Analytical Chemical Adda.

[9] Shetgiri NP, Nayak BK (2005). Indian J Synthesis, antimicrobial and antiinflammatory activities of 1, 3, 4-oxadiazoles linked to naphtho [2, 1-b] furan. Chem.

[10] Ram VJ (1988). Synthesis, spectral, thermal analyses, molecular modeling, and antimicrobial activities of Cu (II)-complexes with 1,3,4-oxadiazole Schiff-base derivatives. Indian J. Chem.

[11] Misra U, Hikari A, Saxena A K, Gurtu S, Shankar K (1996). Synthesis and biological evaluation of some new azolothieno [2,3-d] pyrimidines. Eur. J. Med. C.

[12] Omar F A, Mahfouz N M, Rahman M A, Design (1996). Synthesis and antiinflammatory activity of some 1,3,4-oxadiazole derivatives. Eur. J. Med. Chem.

[13] Mohd Amir, Shikha Kumar (2003). anti‐inflammatory and gastro sparing activity of some new indomethacin derivatives. Indian J. Heterocyclic Chem.

[14] Thomas J, Ger Pat (1961). A anti‐inflammatory and gastro sparing activity of some new indomethacin derivatives. Bstr. Chem.

[15] Hetznein A, Mockel K (1996). Synthesis and antimicrobial activities of oxadiazoles, phthalazines and indolinones. Adv. Hetrocycle Chem.

[16] Sandstorm J (1968). Synthesis and antimicrobial activities of 1,2,4-triazole and 1,3,4-thiadiazole derivatives of 5-amino-2-hydroxybenzoic acid. Adv. Hetrocycle Chem.

[17] Hosam saad (1996). Synthesis and fungicidal activity of 1,3,4-oxadiazole substituted acylthioureas. Indian J. Chem.

[18] Hui XP, Chu, CH, zangzu, wang Q, ZhangQ (2002). Synthesis and characterization of new series of imidazolidin-2,4-dione derivatives and its antimicrobial activity. Indian J. Chem.

[19] Golgolab H, Lalezari I, Hosseini L, Gohari (1973). Synthesis, characterization and anti‐inflammatory activity of 5‐{[((5‐Substituted‐aryl)‐1,3,4‐thiadiazol‐2‐yl) thio]‐n‐alkyl}‐1,3,4‐oxadiazole‐2‐thiol. J. Hetrocycle. Chema.

[20] CAWinter EA, Risely NDG, Nuss N (1962). Carrageenean induced oedema inhind paw of the rat on assay of anti-inflammatorydrugs. Proc. Soc. Exp. Biol.

